# Intensity-Assisted ICP for Fast Registration of 2D-LIDAR

**DOI:** 10.3390/s19092124

**Published:** 2019-05-08

**Authors:** Yingzhong Tian, Xining Liu, Long Li, Wenbin Wang

**Affiliations:** 1 School of Mechatronic Engineering and Automation, Shanghai University, Shanghai 200072, China; troytian@shu.edu.cn (Y.T.); liuxining@shu.edu.cn (X.L.); lil@shu.edu.cn (L.L.); 2 Shanghai Key Laboratory of Intelligent Manufacturing and Robotics, Shanghai University, Shanghai 200072, China; 3 School of Mechanical and Electrical, Shenzhen Polytechnic, Shenzhen 518055, China

**Keywords:** iterative closest point, SLAM, best initial transformation guess, intensity residuals, anderson acceleration

## Abstract

Iterative closest point (ICP) is a method commonly used to perform scan-matching and registration. To be a simple and robust algorithm, it is still computationally expensive, and it has been regarded as having a crucial challenge especially in a real-time application as used for the simultaneous localization and mapping (SLAM) problem. For these reasons, this paper presents a new method for the acceleration of ICP with an assisted intensity. Unlike the conventional ICP, this method is proposed to reduce the computational cost and avoid divergences. An initial transformation guess is computed with an assisted intensity for their relative rigid-body transformation. Moreover, a target function is proposed to determine the best initial transformation guess based on the statistic of their spatial distances and intensity residuals. Additionally, this method is also proposed to reduce the iteration number. The Anderson acceleration is utilized for increasing the iteration speed which has better ability than the Picard iteration procedure. The proposed algorithm is operated in real time with a single core central processing unit (CPU) thread. Hence, it is suitable for the robot which has limited computation resources. To validate the novelty, this proposed method is evaluated on the SEMANTIC3D.NET benchmark dataset. According to comparative results, the proposed method is declared as having better accuracy and robustness than the conventional ICP methods.

## 1. Introduction

The estimation step carried out simultaneously to maintain the robot pose is regarded as the main work of simultaneous localization and mapping (SLAM) [1]. The iterative closest point (ICP) [2] is a well-known algorithm to deal with the laser scanner data presented in the form of point clouds. For example, an improved probabilistically-motivated maximum likelihood estimation (MLE) algorithm including the ICP algorithm was used in [3] to adaptively decrease the search scope in an efficient real-time mobile unmanned ground vehicle (UGV) indoor positioning system for large-area applications. A vehicle localization method based on a vertical corner feature used ICP algorithm to deal with the geometric relations between the scan data of the 3D light detection and ranging (LIDAR) [4]. A technique to estimate the ego motion of a vehicle was presented in [5] where the ICP algorithms were evaluated for their accuracy and computational speed based on laser range data. A 3D mapping system proposed an art ICP-based SLAM method [6] using only point cloud data. A robust and accurate SLAM algorithm for omnidirectional mobile robots based on a novel 2.5D LIDAR device and ICP algorithm was presented in [7,8]. In their works, the 2.5D LIDAR device could also perform vertical scanning within the motion range of the liner stage. Hence, the 2.5D points cloud could be performed by the ICP algorithm to improve the robustness and accuracy of the scan matching. Besides the LIDAR SLAM, the ICP algorithm is also used in the problem of scan-matching in visual SLAM. An adaptation of the ICP algorithm was introduced for the matching of straight lines between two stereo image pairs so that the speed improvement and local minima reduction can be seen in visual SLAM [9]. A real-time, robust, low-drift and depth-only SLAM system applied a local map-based motion estimation strategy using the ICP algorithm to reduce the local drift [10]. 

The concept of the ICP is straightforward. Initially, it estimates the corresponding points between the source and target frame by utilizing a certain metric. Then it estimates the relative transformation with a closed-form solution which aims to minimize the distance between the correspondences. These two steps are iteratively performed until a convergence criterion, or the maximum number, is precisely reached.

There have been many modified ICP algorithms presented nowadays. Globally optimal iterative closest point (Go-ICP) was introduced in [11] to speed up the conventional ICP. Point-to-line metric iterative closest point (PLICP) could converge in a finite number of steps with quadratic speed [12]. A tensor-based ICP did some works on the optimal initial transformation guess [13]. A 3-step approach based on ICP and point Cloud Projection (ICP-CP) performed well for both enhancing the accuracy and reducing the execution in most cases [14]. 4D-ICP was put forward in [15] to accelerate the registration of 3D point cloud segments with hue data from the associated imagery. Besides that, there were lots of variant ICP such as [16,17,18,19].

Intensity information extracted from LIDAR data or visual data sometimes can be used for registration and localization. An intensity-based medical image registration added intensity information into ICP algorithm for scan-matching [20]. The paper [21] put forward a new ICP algorithm assisted with intensity information for visual ranging estimation from a RGB-D camera in visual SLAM. An automatic registration system was presented for aligning intensity scan pairs which was designed to handle several challenges including extensive structural changes, large viewpoint differences, repetitive structure, illumination differences and flat regions [22]. A robust vehicle localization method applied a novel fusion algorithm assisted with vertical and road intensity information for robust localization based on a prior point cloud in urban area [23].

Slightly different from [21] mentioned above, this paper presents a fast and robust ICP based on the assisted intensity method using both the intensity and distance. In addition to adopting the Anderson method encompassed under the used acceleration algorithm, this paper contains the following contributions.

The proposed method utilizing the assisted intensity approach to compute the initial transformation guess of ICP different from that of [21]. The intensity information in [21] is used to be set up as robust weighting function to correct the next transformation in the iterative process with an initial transformation guess still set up as identity matrix. However, the intensity information in this paper is added to a target function to determine the best initial transformation guess based on the statistics of spatial distances and intensity residuals of the point cloud with an iteration process being no robust weighting function.

The rest of this paper is organized as follows. Section 2 describes the conventional ICP method [2] and the Anderson acceleration [24]. The proposed intensity-assisted ICP method is explained in Section 3. Section 4 presents the performance of the proposed method depicted by the evaluation of the SEMANTIC3D.NET benchmark dataset. Finally, a conclusion is presented in Section 5.

## 2. Preliminaries

### 2.1. Conventional Iterative Closest Point (ICP)

The ICP algorithm is commonly used for the robot navigation in performing scan-matching of certain data generated by LIDAR. The source frame *P*_1_ assisted by intensity *I*_1_ can be consider as < *P*_1_, *I*_1_>. Then the target frame is the same as the source frame aligned as < *P*_2_, *I*_2_>. Furthermore the conventional ICP algorithm can be described as follows:

(1) Transform *P*_1_ using initial transformation guess *T*_0_ which is usually set as an identity matrix.

(2) Search for each point in *P*_1_ a closet point in *P*_2_ as correspondence with the transformation*T_k_*_−1_ in the *k*-th iteration of algorithm. For the *i*-th point in *P*_1_, the index of corresponding point in *P*_2_ is described as *index*(*i*), where:

(1) $$index(i)=\underset{j}{\arg\min}\left\|T_{k-1}P_{1}(i)-P_{2}(j)\right\|.$$

(3) Discover transformation *T_k_* that minimizes the distance between the correspondence:

(2) $$T_{k}=\underset{T}{\arg\min}\sum_{i}\left\|TT_{k-1}P_{1}(i)-P_{2}(index(i))\right\|.$$

(4) Apply transformation *T_k_* to *P*_1_.

(5) Stop algorithm when the incremental transformation is smaller than the threshold or oppositely proceed to Step 2.

### 2.2. Anderson Acceleration

The Anderson acceleration [25,26] is a recursive procedure that is usually used to find fixed points of contractive mapping that have been considered to have better performance than conventional picard iteration procedures. Pavlov et al. [24] proposed Anderson accelerated version of ICP (AA-ICP) by utilizing Anderson acceleration. Similarly, Anderson acceleration is also used in this paper to increase the iteration speed to quickly get the final transformation. In the *k*-th iteration, the Anderson acceleration can be described as follows.

(1) As mentioned above in Section 2.1, the process of transformation *T_k_* derived from last transformation *T_k_*_−1_ can be described as a contraction mapping *G*. Thus *T_k_* be described by referring to the following Equation (3):

(3) $$T_{k}=G(T_{k-1}).$$

(2) The error between current transformation *T_k_* and last transformation *T_k_*_−1_ can be described as *ƒ_k_*_−1_. Hence *ƒ_k_*_−1_ can be obtained from Equation (4) below:

(4) $$f_{k-1}=T_{k}-T_{k-1}.$$

(3) The goal of the algorithm is to find α which minimizes,

(5) $$\sum_{j=1}^{n}\alpha_{j}f_{j},$$

where,

(6) $$\sum_{j}\alpha_{j}=1.$$

(4) The optimal transformation *T_k_*_+1_ can be produced by the following Equation (7):

(7) $$T_{k+1}=\sum_{j=1}^{n}\alpha_{j}G(T_{j}).$$

(5) Apply transformation *T_k_*_+1_ into source frame aiming to measure the distance between two frames. Furthermore, the Anderson acceleration will be stopped if the incremental iteration is smaller than a threshold or, by contrast, will proceed to Step 1.

The core content of Anderson acceleration is to minimize iterations as little as simple iterations to blend with the same error. The iteration step cost is neglected compared to a single ICP iteration step while it depends on iteration history. The most important thing is that it can be added trivially to the existing ICP implementation with little modification.

## 3. Intensity-Assisted Iterative Closest Point

The intensity-assisted ICP proposed in this paper is different from the conventional ICP as described below.

(1) **Initial Transformation Guess**—The initial transformation guess is not set as an identity matrix, and determined by utilizing the proposed target function which is based on the statistics of their spatial distances (see detail in Section 2.1) and intensity residuals. It is computed with assisted intensity for their relative rigid-body transformation which aims to reduce the computational cost and avoid divergence.

(2) **Anderson Acceleration**—Anderson acceleration is different from the standard Picard iteration which is commonly used in the conventional ICP. It is used to increase the speed of iteration for quickly reaching a convergence (see details in [24]).

**Figure 1:**
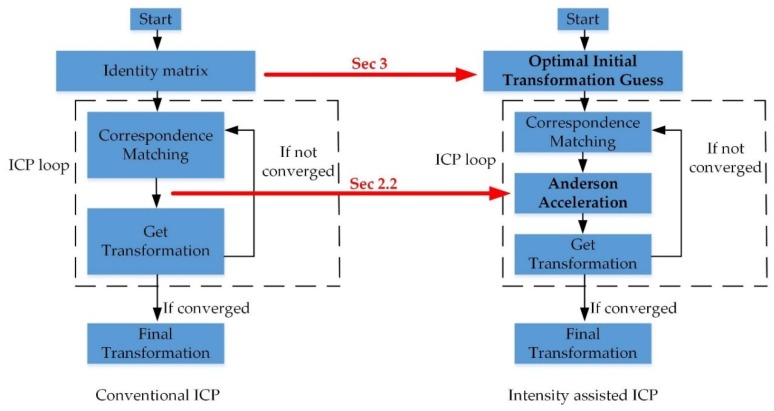
An overview of intensity-assisted iterative closest point (ICP) method compared with conventional ICP.

An overview of the proposed ICP method compared with conventional ICP in this paper can be seen in Figure 1.

As can be observed in the conventional ICP, the initial transformation guess is usually set as identity matrix. This may lead to divergence when the distance of the source and target frame is too large. For this reason, the intensity residuals between correspondences are considered to obtain the optimal initial transformation guess as described below.

### 3.1. Salient Intensity Point Selection

The cost of the computation for the initial transformation may be enormous if there is no sampling process for the point cloud. A new sampling method is proposed in this paper which aims to select the salient point assisted with the intensity that is different from the random sampling method in the point cloud library (PCL).

**Figure 2:**
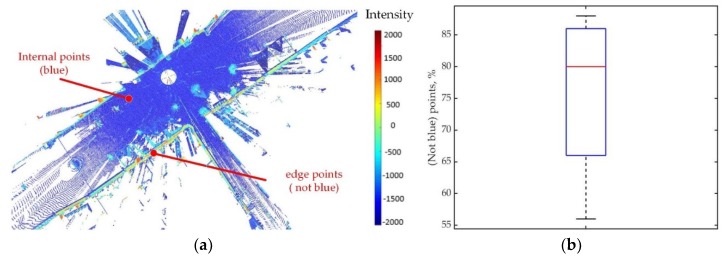
(**a**) Depending on the distribution of intensity value in point cloud, it can be found that the high-intensity points (not blue) are usually on the edge of point cloud with low-intensity points (blue) inside. Therefore, the point cloud composed of high intensity points can represent the shape of the point cloud. (**b**) Based on 100 times experiments to intensity point selection, it can be seen that most of points (not blue) can be extracted for filtered point cloud by Equations (8) and (9).

In this paper, a market square dataset called marketsquarefeldkirch4-reduced that can be found from the SEMANTIC3D.NET benchmark dataset is used in the proposed method. In this dataset, a 2D point clouds of the ground shown in Figure 2 are extracted to support the experiment. The intensity value of the method used to select the salient point is obtained from this dataset. Due to the intensity information in this dataset extracted from 3D Velodyne LIDAR, the method in this paper can perform well in other LIDAR datasets. However, this method may be not suitable for visual point cloud due to the different intensity structure between the LIDAR point cloud and visual point cloud.

As shown in Figure 2a, the intensity value of most of points on the edge of the point cloud is higher than −1000. This is caused by the most points on edge are green points with the intensity of between −1000 and 0. Therefore, the criterion of the salient point x is:

(8) $$-1000<I_{1}(x)<0.$$

(9) $$-1000<I_{2}(x)<0.$$

The filtered point cloud is produced by the random sampling method with this criterion. As can be seen in Figure 2b, the threshold about the −1000 and 0 can be used to obtain 80% of the edge points for filtered point cloud by 100 times the random sampling method. This can prove the validity of this threshold.

**Figure 3:**
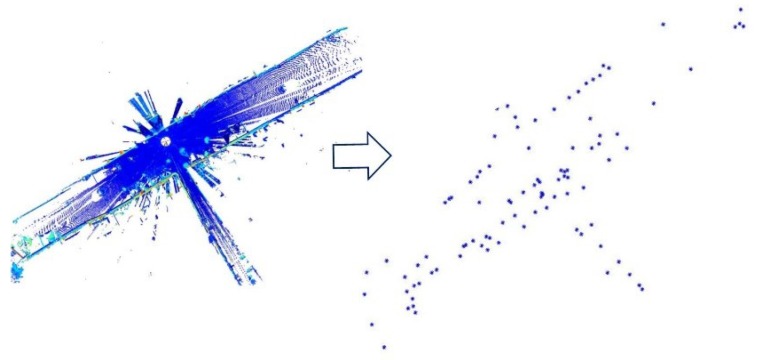
It can be seen that some high intensity points on the edge of the point cloud can be extracted for next registration without so much cost of computation using the method in Section 3.1.

Besides that, the number of the selected points is set to 100 reference to [21] so that it can be used to reduce the cost of computation but not affect the accuracy of the experimental results. Thus, the point cloud filtered by the new sampling method presented in this paper is depicted in Figure 3.

As can be seen, the selected points are usually located on the edge of the point cloud with the internal points having disappeared. By using sampling method mentioned above, the filtered point cloud can represent the direction and shape of the point cloud. Then the filtered point cloud is set up as the source frame and transformed to the target frame with a certain number of candidates of initial transformation guess. Once the filtered point cloud is chosen, it is used for all candidates of initial transformation guess. When the optimal initial transformation guess is obtained, the point cloud for later iteration is the original point cloud but not the filtered point cloud.

### 3.2. Discover Optimal Initial Transformation Guess

In this step, a certain amount of initial transformation guesses {*u*_1_, *u*_2_ *u*_3_, *u*_4_,…, *u_n_*} will be produced by the algorithm. Then, the correspondences between filtered source frame and filtered target frame is established by each initial transformation guess *u_i_*.

Despite the sampling method to choose the points randomly, it can generally cover the entire edge of the point cloud with the criterion in experiment as described in Figure 3. With that the filtered source frame and filtered target frame have the same direction and shape. Hence, a proper transformation can be obtained.

A target function is proposed to determine the best initial transformation guess *u_optimal_* based on statistics of their spatial distances and intensity residuals. Intensity information can be demonstrated so that it can be used to registration and localization as mentioned in [20,22,23].

For example, the corresponding point of *i*-th point in source frame *P*_1_(*i*) is *P*_2_(*index*(*i*)) with an initial transformation guess *u_i_*_._ The intensity residuals and spatial distances between the correspondences are considered for the target function used in this experiment. The intensity residuals *r_i_* ^(*I*)^ of the *i*-th pair are calculated as:

(10) $$r_{i}{}^{\left(I\right)}=I_{1}(i)-I_{2}(index(i)).$$

Inspired by [21,27], the Student’s *t*-distribution is used to scale each intensity residual. The conventional method uses the *t*-distribution to reduce the outliner influence and to determine the correspondence correctly in [21]. By contrast, the use of *t*-distribution in this paper focuses on the computation of its variance which is obviously different from the conventional method. By referring [28], the intensity component of the target function is derived based on *t*-distribution. Thus, weight function *w_I_*(*r_i_*^(*I*)^) is:

(11) $$w_{I}(r_{i}{}^{\left(I\right)})=\frac{v_{\left(I\right)}+1}{v_{\left(I\right)}+{\left(\frac{r_{i}{}^{\left(I\right)}}{\sigma^{\left(I\right)}}\right)}^{2}}.$$

In this experiment, the degree of freedom (DoF) *ν*_(*I*)_ is set to 2.055 and the initial standard variance *σ*^(*I*)^ is set to 7.7189. Notice that the *ν*_(*I*)_ and *σ*^(*I*)^ come from the “fitdist” function in MATLAB. Although *ν*_(*I*)_ and *σ*^(*I*)^ are based on current dataset, the value can still be applied to other Lidar datasets (see Table 1).

By contrast with the usage in [27], the variance of *t*-distribution used in this experiment is used to determine the best initial transformation guess. When a correspondence between the filtered source frame and filtered target frame is established by an initial transformation guess, the *score*^(*I*)^ is computed by using Equation (12):

(12) $$score^{\left(I\right)}=\frac{1}{n}{\sum_{i}(r_{i}}^{\left(I\right)}{)}^{2}w_{I}(r_{i}{}^{\left(I\right)}).$$

**Figure 4:**
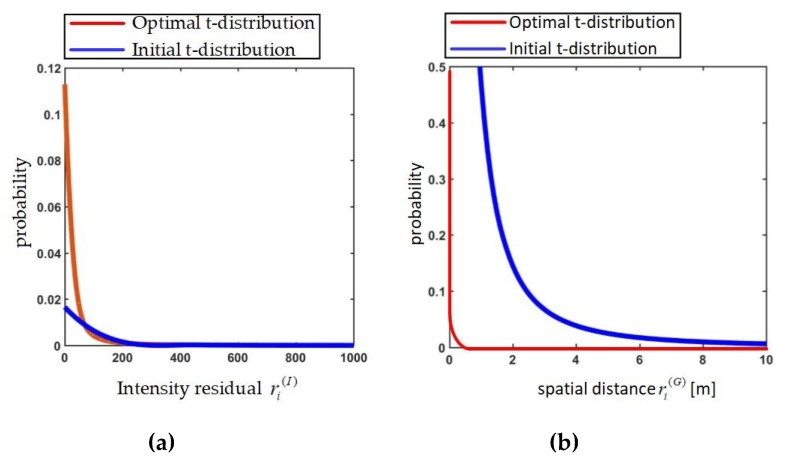
(**a**) The variance of optimal t-distribution about the intensity residuals is lower than that given by initial t-distribution. (**b**) The variance of optimal t-distribution about the spatial distances is lower than that given by initial t-distribution.

The *score*^(*I*)^ is the same as the variance of *t*-distribution. The effect of this score is illustrated in Figure 4a where the variance of optimal *t*-distribution produced by *u_optimal_* is lower than the initial *t*-distribution produced by *u*_1_. It means that most of values of the intensity residuals are zero when optimal initial transformation guess is given. The optimal initial transformation guess can be found in the search process when the *score*^(*I*)^ reaches minimum.

Another component of the target function is based on the spatial distances between correspondences. The spatial distance *r_i_*^(*G*)^ is considered as follows:

(13) $$r_{i}{}^{\left(G\right)}=\left\|T_{k-1}P_{1}(i)-P_{2}(index(i))\right\|.$$

The computation of *w_G_*(*r_i_*^(*G*)^) follows the same method for the intensity residual.

(14) $$w_{G}(r_{i}{}^{\left(G\right)})=\frac{v_{\left(G\right)}+1}{v_{\left(G\right)}+{\left(\frac{r_{i}{}^{\left(G\right)}}{\sigma^{\left(G\right)}}\right)}^{2}}.$$

The degrees of freedom (DoF) of the spatial distances *ν*_(*G*)_ is set to 1.22 which is different from *ν*_(*I*)_. The initial standard variance *σ*^(*G*)^ is set to 0.5326. Then, the *score*^(*G*)^ can be calculated by Equation (15):

(15) $$score^{\left(G\right)}=\frac{1}{n}{\sum_{i}(r_{i}}^{\left(G\right)}{)}^{2}w_{G}(r_{i}{}^{\left(G\right)}).$$

The effect of the *score*^(*G*)^ can be described the same as *score*^(*I*)^. When the optimal *t*-distribution of spatial distance is obtained by utilizing optimal initial transformation guess, the variance is close to zero as can be seen in Figure 4b.

Referring to Figure 4a,b, the variance of t-distribution fits the real data significantly. Then the *score*^(*I*)^ and *score*^(*G*)^ are all normalized for the same weight by Equation (16).

(16) $$SCORE_{u_{i}}^{{}^{\left(I\right)}}=\frac{score_{u_{i}}^{{}^{\left(I\right)}}}{\sum_{n=1}^{n}score_{u_{i}}^{{}^{\left(I\right)}}},SCORE_{u_{i}}^{{}^{\left(G\right)}}=\frac{score_{u_{i}}^{{}^{\left(G\right)}}}{\sum_{n=1}^{n}score_{u_{i}}^{{}^{\left(G\right)}}}.$$

**Figure 5:**
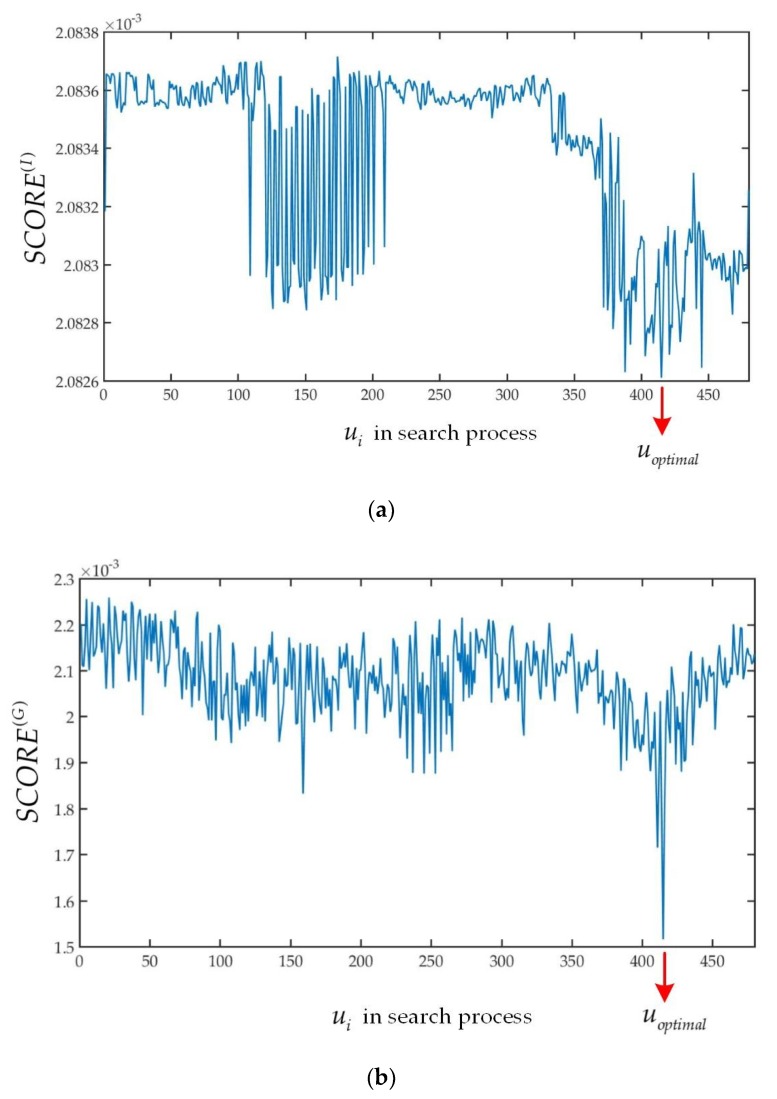
(**a**) The *SCORE*^(*I*)^ curve will reach the minimum when optimal initial transformation guess is used. (**b**) The *SCORE*^(*G*)^ curve will reach the minimum when optimal initial transformation guess is used.

Thus, the curves of the *SCORE*^(*I*)^ and *SCORE*^(*G*)^ in the search process can be depicted by Figure 5a,b, respectively. The *SCORE*^(*I*)^ will be extremely close to the minimum the same as *SCORE*^(*G*)^ when optimal initial transformation guess is given. However, *SCORE*^(*I*)^ and *SCORE*^(*G*)^ may not necessarily sync to the minimum which means that the *SCORE*^(*I*)^ has multiple minimums. The reason is that the correspondence may be identified when a part of the initial transformation guess is close to the given optimal value. Thus the *SCORE*^(*I*)^ computed by optimal initial transformation guess may be the same as others around the optimal value.

However, the *SCORE*^(*I*)^ can help to find the optimal initial transformation guess due to it being able to access to the area including the optimal value. Then the corresponding *SCORE*^(*G*)^ for the same *SCORE*^(*I*)^ are different. By combined with *SCORE*^(*G*)^, the target function that used to find the optimal initial transformation guess *u_optimal_* is described as follows:

(17) $$u_{optimal}=\underset{u_{1},u_{2}\ldots u_{n}}{\arg\min}(SCORE_{u_{i}}^{{}^{\left(I\right)}}SCORE_{u_{i}}^{{}^{\left(G\right)}}).$$

With the new method which is assisted by the intensity, the optimal initial transformation guess can be quickly obtained so that the proceeding speed of ICP will be increased.

Besides the method to compute the initial transformation guess, the Anderson acceleration is also used to increase the speed of iteration. Therefore, the entire algorithm of ICP assisted by the intensity method is represented by Algorithm 1.


**Algorithm 1: Intensity-Assisted ICP**
**Input:** Source frame <*P*_1_, *I*_1_> and target frame <*P*_2_, *I*_2_>, the search criteria *C*_θ,_
*C_x_*, *C_y_*, the search step *S*_θ,_
*S_x_*, *S_y_* **Output**: Convergence transformation *T_final_* get sampled point cloud from *P*_1_, *P*_2_ with Section 3.1 get *C*_θ_, *C_x_*, *C_y_*, *S*_θ_, *S_x_*, *S_y_* with Algorithm 2 *n* = 1 **for** θ = −*C*_θ_: *S*_θ:_
*C*_θ_
**do** **for**
*x* = −*C_x_*: *S_x_*_:_
*C_x_*
**do**  **for**
*y* = −*C_y_*: *S_y_*_:_
*C_y_*
**do**  get the *SCORE*^(*I*)^ and *SCORE*^(*G*)^ with u_n_ produced by θ, *x*, *y*  *n*++  **end for** **end for** **end for** get *u_optimal_* with Section 3.2 *T*_0_ = *u_optimal_* Anderson acceleration begin with *T*_0_ between *P*_1_, *P*_2_ when convergence criteria is true, return *T_final_*

In this algorithm, it is set that *C*_θ_ = 45°, *C_x_* = 1, *C_y_* = 1, *S*_θ_ = 3, *S_x_* = 0.2, *S_y_* = 0.2 for large distances registration with *u_max_* = *u*_3000_ or *C*_θ_ = 10°, *C_x_* = 0.2, *C_y_* = 0.2, *S*_θ_ = 1, *S_x_* = 0.04, *S_y_* = 0.04 for small distance registration with *u_max_* = *u*_2000_. An adaptive algorithm is introduced in this paper to select the thresholds above automatically so that the intensity-assisted ICP can perform well in continuous registration. The process to choose the thresholds is shown below.

In this step, the filtered point clouds obtained in Section 3.1 are used for adaptive selection of thresholds. Inspired by [16], the mean µ and the covariance Σ of filtered point clouds *P* is produced through Gaussian distribution as follows:

(18) $$\mu =\frac{1}{\left|P\right|}\sum P(i),\sum =\frac{1}{\left|P\right|}\sum \left(P(i\right)-\mu {)}^{T}(P(i)-\mu ).$$

Then the covariance Σ is used to obtain eigenvector *ξ*_1_, *ξ*_2_ by eigenvalue decomposition as follows.

(19) $$\sum =(\xi_{N},\xi_{L})\left(\begin{array}{cc} \lambda_{N} & 0\\ 0 & \lambda_{L}^{} \end{array}\right){\left(\xi_{N},\xi_{L}\right)}^{T}.$$

Here *λ*_N_ and *λ*_L_ are the eigenvalues of Σ in ascending order, and *ξ*_N_, *ξ*_L_ can represent the axes of the ellipsoid approximating the point distribution. The eigenvector *ξ*_N_ corresponding to the smaller eigenvalue of *ξ*_N_, *ξ*_L_ is the normal vector so that it describes the direction of the point cloud excellently.

Finally threshold selection is done from the filtered source frame *P*_1_ and the filtered target frame *P*_2_ using Equations (20) and (21):

(20) $$\left\|\mu_{1}-\mu_{2}\right\|\leq 0.2.$$

(21) $$\left\|\xi_{1,N}-\xi_{2,N}\right\|\leq 10^{\circ }.$$

**Figure 6:**
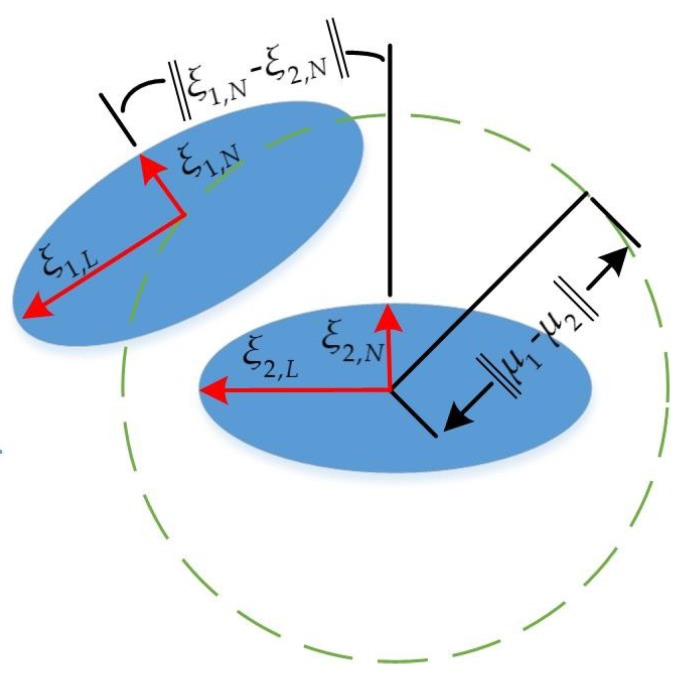
It can be seen that a normal vector can excellently describe the direction of point cloud.

When Equation (20) or Equation (21) is satisfied in algorithm, it is set that *C*_θ_ = 10°, *C_x_* = 0.2, *C_y_* = 0.2 and *S*_θ_ = 1, *S_x_* = 0.04, *S_y_* = 0.04. If not, then the parameters can be set to *C*_θ_ = 45°, *C_x_* = 1, *C_y_* = 1 and *S*_θ_ = 3, *S_x_* = 0.2, *S_y_* = 0.2. The adaptive algorithm to select thresholds is described by Algorithm 2 and Figure 6.


**Algorithm 2: Adaptive Threshold Selection**
**Input:** The filtered source frame *P*_1_ and the filtered target frame *P*_2_ **Output**: The search criteria *C*_θ,_
*C_x_*, *C_y_*, the search step *S*_θ,_
*S_x_*, *S_y_* get *µ*_1_, *µ*_2_, *ξ*_1,N_, *ξ*_2,N_ with Equations (18) and (19) **if** satisfy with Equation (20) || satisfy with Equation (21) **then** *C*_θ_ = 10°, *C_x_* = 0.2, *C_y_* = 0.2 and *S*_θ_ = 1, *S_x_* = 0.04, *S_y_* = 0.04 **else** *C*_θ_ = 45°, *C_x_* = 1, *C_y_* = 1 and *S*_θ_ = 3, *S_x_* = 0.2, *S_y_* = 0.2 **end if** **return** the *C*_θ,_
*C_x_*, *C_y_* and *S*_θ,_
*S_x_*, *S_y_*

## 4. Experimental Results

The proposed intensity assisted ICP algorithm is evaluated using a new large-scale point cloud classification benchmark called SEMANTIC3D.NET [29]. This data set means as for data-hungry (deep) learning methods with over four billion manually labelled points. The SEMANTIC3D.NET benchmark dataset consists of dense point clouds acquired with static terrestrial laser scanners. It contains eight semantic classes and covers a wide range of urban outdoor scenes: churches, streets, railroad tracks, squares, villages, soccer fields and castles, providing more dense and complete point clouds with a much higher overall number of labelled points compared to those already available to the research community.

Due to our new ICP method containing the intensity information, the SEMANTIC3D.NET benchmark dataset is suitable for our experiment compared to other data sets.

In this paper, the proposed method focus on 2D datasets produced from the SEMANTIC3D.NET benchmark dataset, however, it may perform well on 3D ICP as well.

To validate the performance of the proposed method, it was compared with the conventional ICP in point cloud library (PCL). This comparison was initiated by performing both mentioned methods on a desktop computer with Ubuntu 16.04, equipped with Intel Core i7-6700HQ CPU (2.6 GHz) and 16GB RAM. Note that this implementation was only run on a single CPU thread.

All subsequent experiments are based on a dataset called marketsquarefeldkirch4-reduced in the semantic-8 benchmark (contains approximately 10,538,633 points). The part of this point cloud is selected where *z* is less than 2 m and also mapped to the *x*-*y* plane for the experiments.

**Figure 7:**
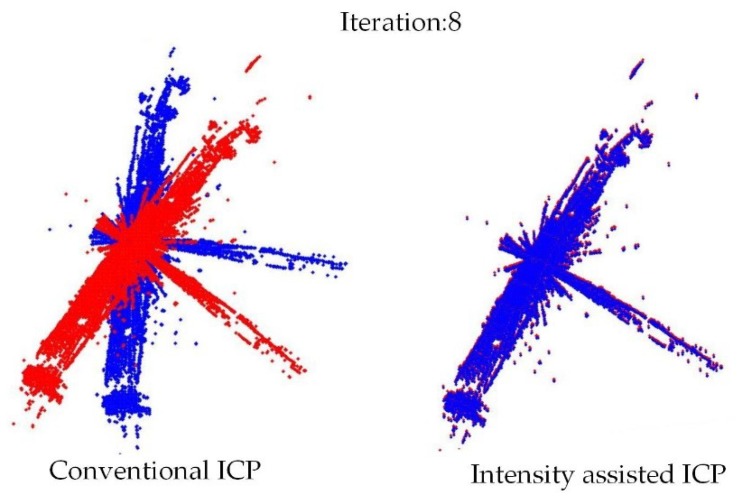
Visual comparison of convergence speed for conventional ICP and intensity-assisted ICP. The blue point cloud shows the source frame taken under 0° and red target frame under 30°. On the 8th iteration intensity-assisted ICP has converged but conventional ICP has not.

**Figure 8:**
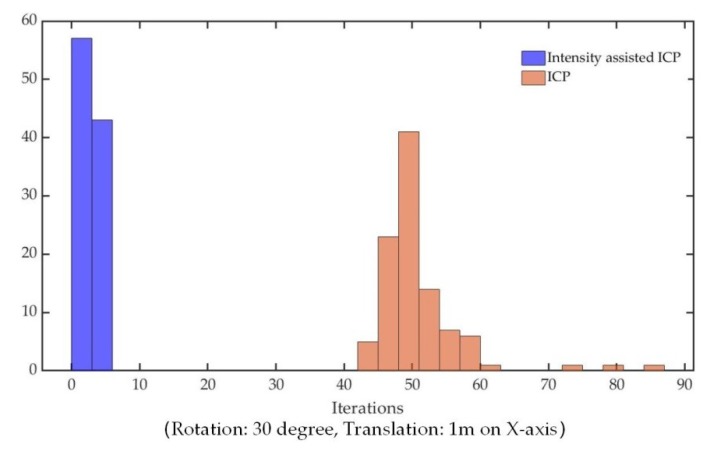
The number of iterations required for intensity-assisted ICP and conventional ICP to converge.

The iteration examples of the intensity-assisted ICP and conventional ICP in PCL are shown in Figure 7 and Figure 8. Based on these figures, it can be shown that the properties of the accelerative statistics for the number of iterations required for convergence is calculated for ε = 0.001.

Note that the experimental data (containing approximately 20,000 points) are taken randomly about 100 times from the original dataset with the PCL random sample method because the original dataset is too large for ICP operation. After each sampling, the obtained point cloud is used as the source frame, and then the point cloud is transformed as the target frame with rotation: 30°, translation: 1 m on the *X*-axis.

Due to *C_x_* = *C_y_* = 1 and *S_x_* = *S_y_* = 0.2, the experimental result (rotation: 30°, translation: 1 m on the *Y*-axis) is the same as that (rotation: 30°, translation: 1 m on the *X*-axis). Hence, the experiments in this paper are focused on the situation (rotation: 30°, translation: 1 m on the *X*-axis).

As illustrated by Figure 8, the number of iterations of the proposed method is clearly reduced compared to conventional ICP. That is because of the optimal initial transformation guess and Anderson acceleration.

**Figure 9:**
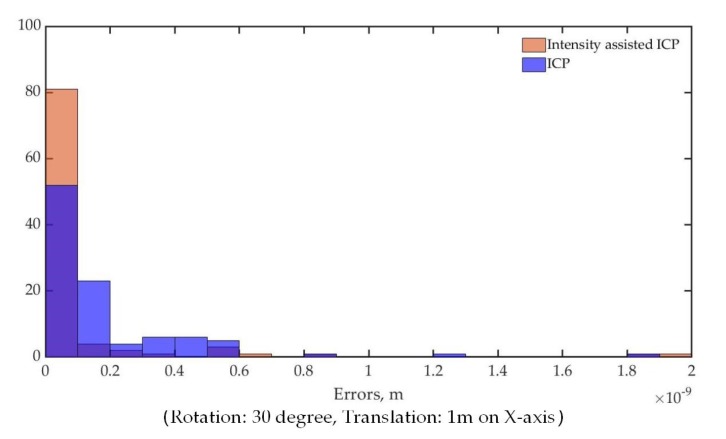
The final error convergence of intensity-assisted ICP and conventional ICP.

In addition to the comparison of iterations for convergence, intensity-assisted ICP generates some results with the same quality as given by conventional ICP or even better. This can be seen from Figure 9, which depicts the error distribution of intensity-assisted ICP and conventional ICP. The error is estimated as the sum of the differences between correspondences in an Euclidean sense, divided by the number of correspondences. Most of runs converge to the same errors, but the number of errors produced from intensity-assisted ICP distributed between 0 and 1 × 10^−10^ are more than that from conventional ICP, which means that intensity-assisted ICP may be more robust than conventional ICP.

**Figure 10:**
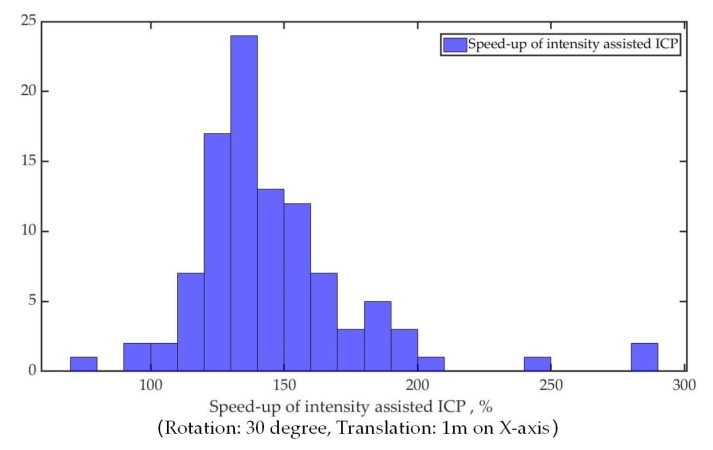
The intensity-assisted ICP speed-up relative to the conventional ICP.

Due to the process to produce a certain amount of the initial transformation guess, the reduction in number of iterations may not prove the efficiency of the proposed method. The relative change of speed to converge between two algorithms for the same dataset is shown in Figure 10. The speed is calculated from the running time of two algorithms on the same computer. Figure 10 can demonstrate that although the process used to generate the initial transformation guess takes a certain amount of time, the average speed of the intensity-assisted ICP method is still faster than the conventional ICP.

**Figure 11:**
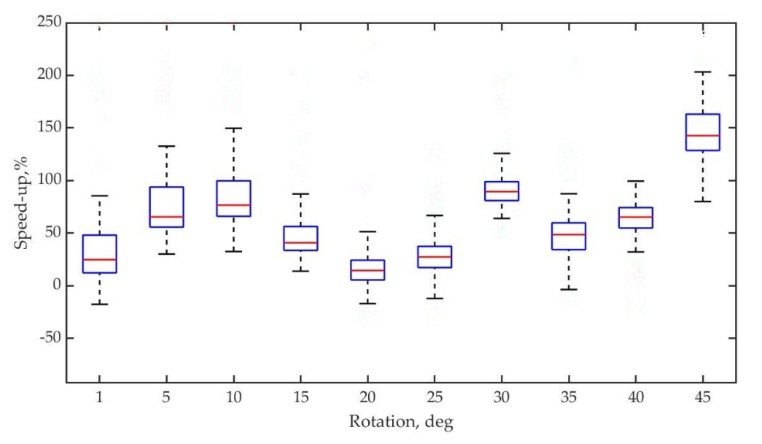
Box-plot of relative intensity-assisted ICP speed-up versus random rotation angle for dataset.

To test the acceleration properties of intensity-assisted ICP, 100 random iterations per given degree are introduced and then the relative acceleration is recorded and compared to conventional ICP, where ε is calculated as 0.001. As can be seen, Figure 11 illustrates the result of the experiments. Relative speed-up of intensity assisted ICP is all higher than that of conventional ICP when rotation increases and finally stay on the slope of approximately 150% when rotation = 45°.

**Figure 12:**
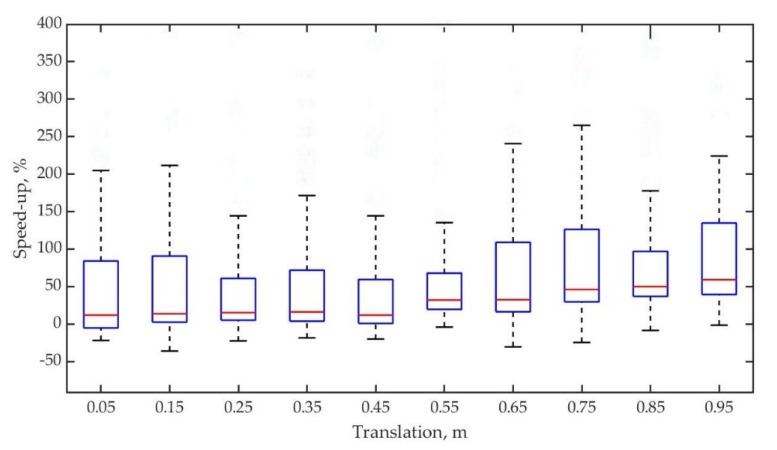
Box-plot of relative intensity assisted ICP speed-up versus random translation distance for dataset.

The next experiment was conducted for translations. Similarly, 100 random translations per given distance were also introduced. As demonstrated in Figure 12, it looks quite similar to the experiments with random rotations. Relative speed-up of intensity-assisted ICP is all higher than that of conventional ICP when distance increases and can stay on the slope of approximately 50% when translation = 0.95 m.

Note that the large fluctuation of relative speed-up originates from different point clouds per sampling operation.

**Figure 13:**
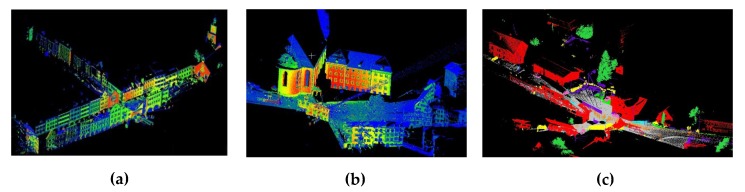
(**a**) The Market datasets (marketsquarefeldkirch4-reduced). (**b**) The Stgallen datasets (stgallencathedral6-reduced). (**c**) The Station datasets (untermaederbrunnen1).

Finally the intensity-assisted ICP is compared with conventional ICP, AA-ICP and implicit moving least squares-ICP (IMLS-ICP) [30] with datasets Market (marketsquarefeldkirch4-reduced), Stgallen (stgallencathedral6-reduced), Station (untermaederbrunnen1) extracted from SEMANTIC3D.NET using the downsampling method with the sampled grid size 0.1 m different from the rand sampling method above. The three original datasets can be seen in Figure 13.

As shown in Table 1, the intensity-assisted ICP performs well in the three datasets even better than experiments above. The reason is that the sampling method is downsampling in this step but in the experiments above it is rand sampling. The intensity-assisted ICP can achieve faster speed and better accuracy compared to conventional ICP and AA-ICP. The IMLS-ICP algorithm [30] is a state-of-the-art ICP algorithm introduced in 2018 and ranked third in the KITTI dataset. The remarkable feature of the IMLS-ICP algorithm is its high accuracy. The intensity-assisted ICP is also faster to converge than IMLS-ICP but with low accuracy. This may be a disadvantage of intensity-assisted ICP, but the intensity-assisted ICP can still maintain excellent performance compared to other algorithms.

## 5. Conclusions

As discussed above, the intensity-assisted ICP is proposed and analyzed. A novel modification of the conventional ICP based on the initial transformation guess and Anderson acceleration indicates the proposed method can perform well in this paper. On the initial transformation guess stage, this new method improves the conventional ICP significantly. A target function is proposed to determine the best initial transformation guess based on the statistic of their spatial distances and intensity residuals with a little runtime cost. It reduces substantially the number of iterations required for achieving desired matching quality compared to the conventional ICP. Besides that, the Anderson acceleration is also used to increase the speed of iteration which has been considered as having better performance compared to the conventional Picard iteration procedure. Moreover, the SEMANTIC3D.NET benchmark dataset is used to evaluate the proposed method. The experimental results show that the new method presented in this paper achieves better speed and quality for convergence compared to the conventional ICP.

## Figures and Tables

**Table 1:** Comparison of the speed and accuracy of four ICP algorithms.

Dataset	Parameters	Algorithms			
		ICP	AA-ICP	IMLS-ICP	Our ICP
Market	Experiment1 (1 m, 0 m, 30°)				
	Errors (m)	5.808 × 10^−3^	3.644 × 10^−3^	1.317 × 10^−4^	1.556 × 10^−^^3^
	Time (s)	125.936	76.455	45.469	12.674
	Experiment2 (0.5 m, 0 m, 15°)				
	Errors (m)	5.806 × 10^−3^	3.261 × 10^−3^	3.746 × 10^−^^5^	2.185 × 10^−3^
	Time (s)	56.454	23.113	42.924	13.901
	Experiment3 (0.1 m, 0 m, 10°)				
	Errors (m)	1.755 × 10^−3^	1.757 × 10^−3^	3.293 × 10^−^^4^	1.659 × 10^−9^
	Time (s)	54.458	37.008	48.154	22.093
Stgallen	Experiment1 (1 m, 0 m, 30°)				
	Errors (m)	8.662 × 10^−3^	5.891 × 10^−3^	1.523 × 10^−^^4^	3.265 × 10^−3^
	Time (s)	239.642	168.634	138.276	21.272
	Experiment2 (0.5 m, 0 m, 15°)				
	Errors (m)	6.039 × 10^−3^	1.971 × 10^−3^	6.603 × 10^−^^5^	1.853 × 10^−3^
	Time (s)	158.853	70.818	121.159	23.416
	Experiment3 (0.1 m, 0 m, 10°)				
	Errors (m)	4.159 × 10^−3^	9.012 × 10^−3^	4.203 × 10^−^^5^	1.942 × 10^−3^
	Time (s)	156.046	59.321	114.177	20.718
Station	Experiment1 (1 m, 0 m, 30°)				
	Errors (m)	3.096 × 10^−2^	1.527 × 10^−2^	2.417 × 10^−^^5^	2.357 × 10^−3^
	Time (s)	57.688	47.728	56.993	12.524
	Experiment2 (0.5 m, 0 m, 15°)				
	Errors (m)	1.368 × 10^−2^	1.079 × 10^−2^	2.387 × 10^−^^4^	9.748 × 10^−4^
	Time (s)	51.574	37.425	64.251	12.576
	Experiment3 (0.1 m, 0 m, 10°)				
	Errors (m)	4.911 × 10^−3^	8.655 × 10^−3^	9.817 × 10^−^^5^	1.049 × 10^−4^
	Time (s)	54.861	41.817	42.546	12.876
